# Lactation Defect in a Widely Used MMTV-Cre Transgenic Line of Mice

**DOI:** 10.1371/journal.pone.0019233

**Published:** 2011-04-29

**Authors:** Taichang Yuan, Yongping Wang, Lily Pao, Steve M. Anderson, Haihua Gu

**Affiliations:** 1 Department of Pathology, University of Colorado School of Medicine, University of Colorado, Aurora, Colorado, United States of America; 2 Cancer Biology Program, Division of Hematology and Oncology, Department of Medicine, Beth Israel and Deaconess Medical Center and Harvard Medical School, Boston, Massachusetts, United States of America; Garvan Institute of Medical Research, Australia

## Abstract

**Background:**

MMTV-Cre mouse lines have played important roles in our understanding about the functions of numerous genes in mouse mammary epithelial cells during mammary gland development and tumorigenesis. However, numerous studies have not included MMTV-Cre mice as controls, and many investigators have not indicated which of the different MMTV-Cre founder lines were used in their studies. Here, we describe a lactation defect that severely limits the use of one of the most commonly used MMTV-Cre founder lines.

**Methodology/Principal Findings:**

To explore the role of protein tyrosine phosphatase Shp1 in mammary gland development, mice bearing the floxed Shp1 gene were crossed with MMTV-Cre mice and mammary gland development was examined by histological and biochemical techniques, while lactation competency was assessed by monitoring pup growth. Surprisingly, both the Shp1fl/+;MMTV-Cre and MMTV-Cre female mice displayed a severe lactation defect when compared to the Shp1 fl/+ control mice. Histological and biochemical analyses reveal that female mice expressing the MMTV-Cre transgene, either alone or in combination with floxed genes, exhibit defects in lobuloalveolar expansion, presence of large cytoplasmic lipid droplets in luminal alveolar epithelial cells postpartum, and precocious induction of involution. Using a PCR-based genotyping method, the three different founder lines can be distinguished, and we determined that the MMTV-Cre line A, the most widely used MMTV-Cre founder line, exhibits a profound lactation defect that limits its use in studies on mammary gland development.

**Conclusions/Significance:**

The identification of a lactation defect in the MMTV-Cre line A mice indicates that investigators must use MMTV-Cre alone mice as control in studies that utilize Cre recombinase to excise genes of interest from mammary epithelial cells. Our results also suggest that previous results obtained in studies using the MMTV-Cre line A line should be re-evaluated if the controls did not include mice expressing only Cre recombinase.

## Introduction

The ability to excise specific genes in a tissue-specific manner has offered a great advance in our ability to determine the roles that specific genes play in development and diseases such as cancer at the level of the whole animal [1], [2]. The current technology involves the excision of a gene locus that is flanked by *loxp* sites (floxed) through the expression of bacterial-derived Cre recombinase in an organ or cell-specific manner through the use of tissue-specific promoters [3]. The wide use of this technology requires the generation of numerous transgenic mouse lines in which the Cre recombinase gene is expressed under the control of different tissue specific promoters; the current mouse database from The Jackson Laboratories lists 268 unique entries for transgenic mice expressing Cre recombinase (www.jax.org) demonstrating the widespread interest in the use of this technology. These Cre-expressing strains play important roles in understanding the functions of genes in normal developmental processes and neoplastic progression. Although many studies involving tissue specific deletion of genes using the Cre-*loxp* recombination technology have analyzed control mice that express Cre recombinase in the absence of floxed genes, there are numerous published studies in which the only control animals used were those homozygous for the presence of the floxed alleles, and the Cre-expressing mice were not used as control animals even though toxic effects of Cre have been observed when it is expressed in mammalian cells alone [4].

Analysis of the contribution of specific genes in mammary gland development and tumorigenesis has been aided by the use of tissue specific promoters to overexpress genes of interest in the mammary gland, and these promoter systems have been extended to the tissue-specific expression of Cre recombinase. Three different promoter systems have been extensively used for studies on the mammary gland including MMTV-Cre (mouse mammary tumor virus ) [5], [6], [7], [8], WAP-Cre (whey acidic protein) [5], and BLG-Cre (beta-lactoglobulin) [9]. The most recent development has been bicistronic constructs that express both the Neu oncogene and Cre recombinase, MMTV-NIC, which allows for excision of genes of interest in cells that also express the Neu oncogene [10]. Although each of these promoter systems has distinct advantages with regard to tissue-specificity and the temporal pattern of expression, the MMTV-Cre mice, particularly those developed by the Hennighausen laboratory [5], [6], have been perhaps the most extensively used Cre-expressing transgenic mouse lines with regard to the mammary gland. Differences in the pattern of Cre expression have been observed in three different founder lines (A, D, and F) of the MMTV-Cre mice generated by the Hennighausen laboratory. However, in many published papers, investigators have not specifically mentioned which of these different MMTV-Cre mouse lines were used in their studies. Importantly, mammary gland development in these different MMTV-Cre mouse lines during pregnancy and lactation has never been carefully described. We have uncovered a lactation defect in one of the MMTV-Cre founder lines during our study of the effect of the SH2-domain containing protein tyrosine phosphatase1 (Shp1) upon mammary gland development.

Shp1 plays an important role in negatively regulating the growth and differentiation of various hematopoietic cell lineages [11]. In addition to its high expression in hematopoietic cells, Shp1 is expressed in epithelial cells including mammary epithelial and breast cancer cell lines [12]. However, the role of Shp1 in the growth and differentiation of mammary epithelial cells *in vivo* is still not well understood. To explore the function of Shp1 in mammary gland during puberty, pregnancy, and lactation we crossed the Shp1 floxed mice [13] with the MMTV-Cre mice [5], in which Cre is expressed in mammary epithelial cells of both virgin and pregnant mice [6]. Surprisingly, we found that the MMTV-Cre female mice displayed a lactation defect due to impaired lobular-alveolar expansion and differentiation during pregnancy and early lactation. We identified that the MMTV-Cre line in our study was line A, which is one of the most widely used MMTV-Cre lines [6]. Analysis of integrated transgene revealed that the MMTV-Cre transgene is physically linked to the cytokeratin 14 (K14)-agouti transgene [14] in these mice. The results of our study indicate the importance of using Cre mice alone as a control in any studies involving the Cre-*loxp* recombination technology, and also suggest that some MMTV-Cre lines are not appropriate for studies on mammary gland development during pregnancy and lactation, due to a profound lactation defect.

## Results

### Shp1 fl/+;MMTV-Cre and MMTV-Cre female mice have severe lactation defect

To explore the role of protein tyrosine phosphatase Shp1 in mammary gland development, we crossed mice bearing floxed alleles (fl) of Shp1 [13] to the widely used MMTV-Cre mice [6] to obtain the Shp1 fl/+; MMTV-Cre female mice. These mice were then mated to Shp1 fl/+ male mice to generate Shp1 fl/fl;MMTV-Cre female mice. The first litters born by all five of the Shp1 fl/+/;MMTV-Cre female mice were of normal litter size. Surprisingly, none of the pups nursed by the Shp1 fl/+;MMTV-Cre dams gained weight normally over the first ten days of lactation, and all of the pups in four of the five litters died by day eighteen of lactation (L18). The dead pups all appeared to be significantly smaller when compared to pups nursed by normal dams at the same stage of growth. One litter of 8 pups survived until L18; however, the average weight of these pups was 3.9 grams regardless of the genotype or sex of the pup, compared to more than 10 grams observed in normal pups nursed by normal dams at L18. The lack of pup growth during lactation was exhibited by all pups regardless of their sex or genotype, and suggested to us that the Shpi1fl/+;MMTV-Cre dams must display a lactation defect [15].

**Figure 1:**
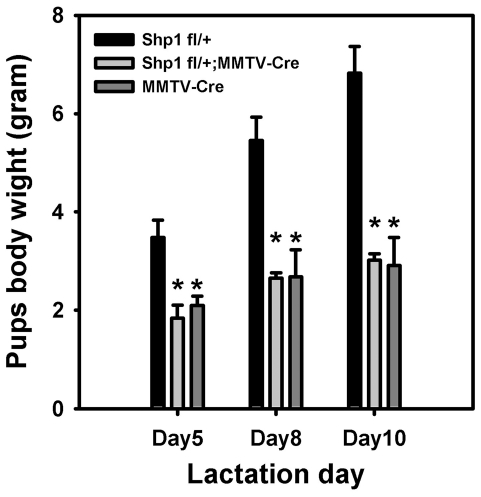
Shp1 fl/+;MMTV-Cre and MMTV-Cre display a lactation defect. Shp1 fl/+ (control), Shp1 fl/+;MMTV-Cre, and MMTV-Cre female mice were mated with wild type FVB male mouse. On the first day following parturition, the litter size was normalized to 6 pups/litter. Pups were weighted at lactation days 5, 8, and 10. The average weight of each litter is shown and bars indicate the standard deviation (SD). ** indicates P<0.001. The data shown is representative of at least two independent experiments.

To further verify that the Shp1 fl/+;MMTV-Cre dams displayed a lactation defect, age-matched virgin Shp1 fl/+;MMTV-Cre female mice and control Shp1 fl/+ female mice were mated with wild type male mice. At L1, both pup and litter sizes appeared to be the same for both the Shp1 fl/+;MMTV-Cre and Shp1 fl/+ dams. Litter size was normalized to six pups per dam as a means to provide equal demand on the mammary gland during lactation, and the pups were then weighed at L5, L8, and L10. Consistent with our initial observation, the weights of the pups nursed by the Shp1 fl/+;MMTV-Cre dams were significantly reduced by 50% or more compared to the weights of control litters, at L5, L8, and L10 (Figure 1). As a second control we also included analysis of litters nursed by MMTV-Cre dams (Figure 1). The weights of pups nursed by MMTV-Cre dams were identical to that observed with pups nursed by the Shp1 fl/+;MMTV-Cre dams; pups nursed by MMTV-Cre dams were 50% smaller than pups nursed by Shp1fl/+ dams at all time points examined. Although the MMTV-Cre mice used in this study were young adults, we have also observed that 6-month-old MMTV-Cre female mice are unable to support the normal growth of their pups (data not shown), suggesting that this lactation defect was not the result of delayed ductal development during puberty. These data indicate that dams carrying the MMTV-Cre transgene exhibit a profound lactation defect.

**Figure 2:**
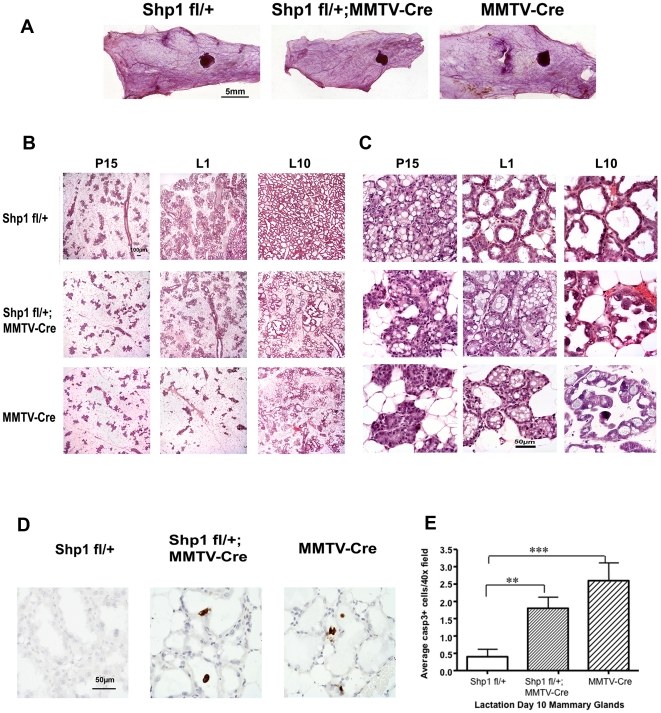
Impaired differentiation and precocious involution of mammary epithelial cells in mice expressing the MMTV-Cre transgene. (A) The number 4 mammary glands from Shp1 fl/+ (control), Shp1 fl/+/;MMTV-Cre, and MMTV-Cre mice were isolated from 10-week old virgin mice and subjected to whole mount analysis as described in the Materials and Methods. Scale bar = 5 mm. (B, C) Six-week old Shp1 fl/+ (control), Shp1 fl/+;MMTV-Cre, and MMTV-Cre female mice were mated with male wild type mice. The number 4 mammary glands were removed at pregnancy day 15 (P15), lactation day 1 (L1), and day 10 (L10), and processed for histological analysis as described in the Materials and Methods. Images were taken under lower (B) and higher (C) magnification respectively. Scale bars represent 100 µm in (B) and 50 µm in (C). Similar results were seen from at least two different mice for each genotype. (D, E) Analysis of apoptotic cells in L10 mammary glands as described in (B, C). (D) Sections of L10 mammary glands from indicated mice were stained with antibody against cleaved caspase 3 (see Materials and Methods). Cells positive for cleaved caspase 3 are stained dark-brown. Images were taken using 40X objective lens. Scale bar = 50 µm. (E) Quantification of caspase 3 positive cells from (D) (see Materials and Methods). Two mice from each genotype were analyzed. **p<0.01 and ***p<0.001.

To further characterize the lactation defects and determine when they occurred during mammary gland development and remodeling, we analyzed the histology of the number four mammary gland at different developmental stages from the Shp1 fl/+;MMTV-Cre, MMTV-Cre, and Shp1 fl/+ female mice. Whole-mount analysis was conducted using mammary glands from the 10-week old virgin mice. Compared to the Shp1 fl/+ control gland, the mammary glands from both Shp1 fl/+;MMTV-Cre and MMTV-Cre mice showed grossly normal ductal elongation, growth and side branching (Figure 2A). We also conducted histological examination of hematoxylin and eosin stained thin sections of the mammary glands from these three lines of mice, during pregnancy and lactation. At pregnancy day 15 (P15) the density of mammary alveoli are similar in the mammary glands from all three lines of mice (Figure 2B). However, when these sections are examined under higher magnification, the mammary epithelial cells in alveoli from the Shp1 fl/+ control mice were noted to contain numerous large cytoplasmic lipid droplets (CLDs). In contrast, mammary alveolar epithelial cells in the Shp1 fl/+;MMTV-Cre and the MMTV-Cre mice have decidedly fewer and smaller CLDs at P15 (Figure 2C). At L1, the lumina of the mammary alveoli from both the Shp1 fl/+;MMTV-Cre as well as and the MMTV-Cre mice had not expanded, and the epithelial cell layer appears to be thinner. The presence of larger mammary adipocytes in mammary glands of both the Shp1 fl/+;MMTV-Cre and MMTV-Cre mice makes it hard to determine whether there are fewer alveolar structures or not (Figure 2B and C). In addition, although the CLDs in the mammary epithelial cells from the Shp1fl/+ mice are small, which is typical of a mouse that has undergone secretory activation, the CLDs in the mammary epithelial cells from both the Shp1 fl/+;MMTV-Cre and the MMTV-Cre mammary glands were larger (Figure 2C). The presence of large CLDs in mammary epithelial cells and the absence of expanded luminal space suggest that the secretory activation has not occurred completely [16].

By L10, the mammary glands from the Shp1fl/+ mice are completely filled with the fully expanded secretory alveoli (Figure 2B), with evidence of milk secretion as exemplified by proteinaceous material in the luminal space (Figure 2C), and the near absence of mammary adipocytes (Figures 2B and C). In contrast, the mammary glands from both the Shp1 fl/+;MMTV-Cre and MMTV-Cre dams still contained a large portion (∼50%) of mammary adipocytes that are readily apparent even at L10 (Figure 2B). Furthermore, detached epithelial cells, which are likely apoptotic cells, were readily apparent at L10 in the luminal space of secretory alveoli from both Shp1 fl/+;MMTV-Cre and the MMTV-Cre mice (Figure 2C). Results from cleaved caspase3 immunostaining confirm that the numbers of apoptotic cells in the L10 mammary glands are increased dramatically in Shp1 fl/+; MMTV-Cre and MMTV-Cre dam compared to control dams (Figure 2D and 2E). These data strongly suggest that the Shp1 fl/+; MMTV-Cre and MMTV-Cre female have defects in secretory activation and functional differentiation of luminal epithelial cells, resulting in the early onset of involution even when the pups are being nursed by these dams. This precocious induction of involution likely further contributes to the observed lactation defect.

### Abnormal activation of Stat5 and Stat3 in the mammary glands of mice expressing the MMTV-Cre transgene

**Figure 3:**
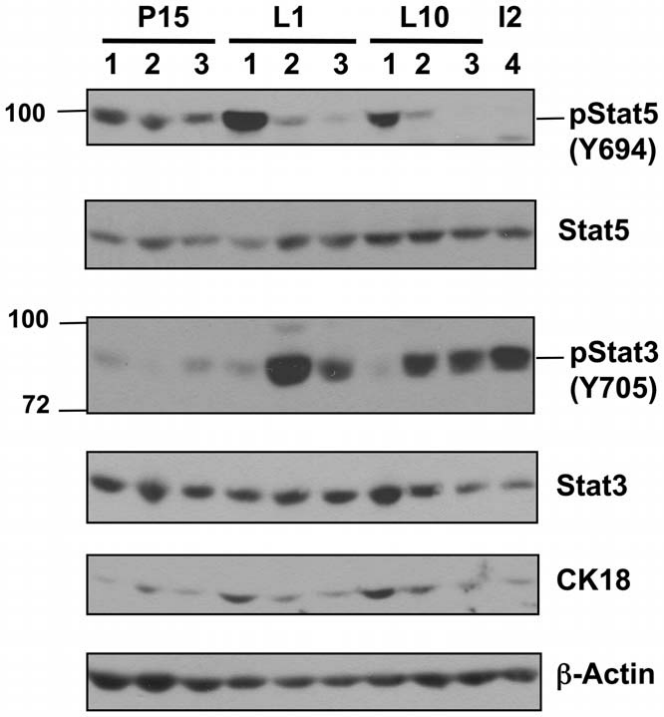
Changes in the activation of Stat5 and Stat3 in mammary glands from mice expressing the MMTV-Cre transgene during late pregnancy and early lactation. The number 4 mammary glands from Shp1 fl/+ (control) (lane 1), Shp1 fl/+;MMTV-Cre (lane 2), and MMTV-Cre (lane 3) mice at pregnancy day 15 (P15), lactation day 1 (L1), day 10 (L10), and from wild type FVB mice at involution day 2 (I2) (lane 4) were isolated and whole tissue lysates prepared as described in the Materials and Methods. Equal amount of proteins (∼40 µg) from each sample were resolved by gel electrophoresis, and immunoblotted with antibodies against phospho-Stat5 (p-Stat5; Y694), or phospho-Stat3 (p-Stat3; Y705). The immunoblots were reprobed with antibodies against Stat5, Stat3, and cytokeratin-18 (CK-18), and beta-actin to demonstrate equal loading. Similar results were seen from at least two different mice for each genotype.

Stat5 activation is required for the proliferation and differentiation of mammary epithelial cells during pregnancy and lactation [17], while activation of Stat3 is required for mammary gland involution [18]. Therefore we examined the activation of both Stat5 and Stat3 by immunoblotting mammary gland lysates from Shp1fl/+, Shp1 fl/+;MMTV-Cre, and MMTV-Cre mice with antibodies that recognize the phosphorylated, activated forms of each Stat protein (Figure 3). At P15, activation of Stat5, as indicated by phosphorylation of tyrosine 694 (Y694), was similar in glands from Shp1fl/+, Shp1 fl/+;MMTV-Cre and MMTV-Cre mice. At L1 robust phosphorylation of Stat5 was observed in the mammary glands from Shp1fl/+ mice (Figure 3, top panel, lane 1), however, phosphorylation of Stat5 was significantly reduced in the mammary glands from Shp1 fl/+;MMTV-Cre and MMTV-Cre mice (Figure 3, top panel, lanes 2 and land 3). At L10, phosphorylation of Stat5 was robust in Shp1fl/+ mammary glands, whereas phosphorylation of Stat5 remained reduced in glands from Shp1 fl/+;MMTV-Cre and MMTV-Cre mice. Conversely, at both L1 and L10, activation of Stat3 was prominent in mammary glands from both Shp1 fl/+;MMTV-Cre (Figure 3, second panel, lane 2) and MMTV-Cre mice (lane 3), compared to the amount of phosphorylated Stat3 in control glands from Shp1fl/+ mice (lane 1). Interestingly, the level of phosphorylated Stat3 observed in the mammary glands from Shp1 fl/+;MMTV-Cre and MMTV-Cre mice at L1 and L10 is comparable to that observed in mammary glands from wild type FVB mice on involution day 2 (lane 4). Thus, both the histological and biochemical data strongly suggest that the MMTV-Cre female mice have impaired functional differentiation of mammary epithelial cells, which when coupled with an early onset of mammary involution, explains why dams that carry the MMTV-Cre transgene are unable to lactate normally. Furthermore these data suggest that the lactation defect observed in the Shp1 fl/+;MMTV-Cre female is not due to the 50% decrease in Shp1 protein expression, but rather due to changes associated with the presence of the MMTV-Cre transgene.

### MMTV-Cre transgene is physically linked to the K14-agouti cassette

**Figure 4:**
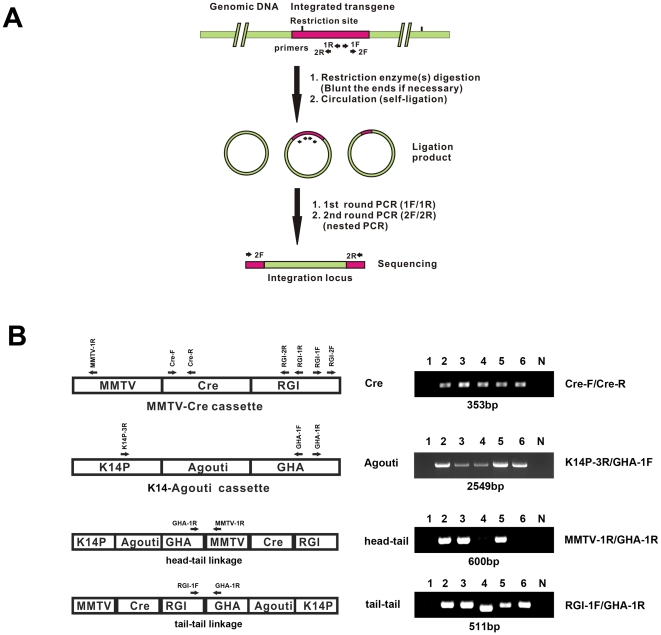
Identification of the linkage of the K14-Agouti cassette to the MMTV-Cre transgene using inverse PCR. (A) The schematic diagram of the inverse PCR. (B) PCR using specific primer sets identified the K14-agouti cassette adjacent to the MMTV-Cre transgene. The linkage of the linkage of the MMTV-Cre and K14-agouti transgenes allow distinction of the line A, line D and line F founder lines. The MMTV-Cre cassette [5] also contains a rabbit growth hormone intron sequence (RGI). The K14-Agouti cassette [14] also contains a human growth hormone polyadenylation sequence (GHA). The Cre-F/Cre-R primer set identifies the presence of Cre gene. The K14P-3R/GHA-1F primer set identifies the presence of the K14-agouti cassette. The MMTV-1R/GHA-1R primer set identifies the presence of the head-to-tail linkage of the K14-agouti cassette and the MMTV-Cre cassette. The RGI-1F/GHA-1R primer set identifies the presence of the tail-to-tail linkage of the MMTV-Cre cassette and the K14-agouti cassette. 1, wild type DNA; 2, MMTV-Cre DNA used in this study; 3, MMTV-Cre line A DNA from Jackson lab; 4, MMTV-Cre line D from Jackson lab; 5, MMTV-Cre line A DNA from Hennighausen lab; 6, MMTV-Cre line F DNA from Hennighausen lab; N, no DNA control. Note, lane 2, 3, and 5 (all Line A) have the same PCR fragments for the head-tail and the tail-tail linkages whereas lane 4 (line D), and lane 6 (line F) only have PCR fragments for the tail-tail linkage. The sizes of PCR products are listed under each DNA gel panel.

The defect in secretory activation and lactation in the MMTV-Cre female mice, developed by the Hennighausen laboratory [5], has not been previously reported in the literature, nor have such developmental defects been described in MMTV-Cre mice developed by the Muller laboratory [7]. Although toxic effects of elevated Cre recombinase expression have been reported [4], we also considered whether there were features unique to the MMTV-Cre lines used in our studies that might underlie the observed lactation defect. One possible explanation is that the lactation defect in this line of MMTV-Cre mice could result from the integration of the MMTV-Cre transgene into a gene critically important for mammary epithelial differentiation, secretory activation, or lactation. To address this possibility, we decide to clone the integration site for the MMTV-Cre transgene using the inverse PCR [19] as outlined in Figure 4A. Briefly, this method involves digestion of genomic DNA with restriction enzymes, followed by ligation of the digested genomic DNA to generate circular DNA molecules. The circular DNAs are subjected to two rounds of PCR amplification using two pairs of nested primers [rabbit growth hormone intron sequence (RGI)-1F/RGI-1R and RGI-2F/RGI-2R] located in the MMTV-Cre transgene (Figure 4A and B) (See Table 1 for primer sequences). Sequencing the DNA product from the second round of PCR amplification should yield information regarding the DNA sequences that are linked to the MMTV-Cre transgene in the mouse genome, and thus represent the integration site.

**Table 1:** Primers used for inverse PCR and genotyping the MMTV-Cre mice.

Primer	Sequence (5′–3′)
RGI-1F	GGTTGTTGTGCTGTCTCATC
RGI-1R	CACGTTGCCCAGGAGCTGTA
RGI-2F	GAAGCCCCTTGAGCATCTGA
RGI-2R	CCCGGTTTGGACTCAGAGTA
MMTV-1R	AGAGGAAGTTGGCTGTGGTC
Cre-F	ATGTCCAATTTACTGACCG
Cre-R	CGCCGCATAACCAGTGAAAC
GHA-1F	CCCTACAGGTTGTCTTCCCA
GHA-1R	TGCTGGGATTACAGGCGTGA
K14P-3R	GGACCAAGTGGACTCCTAGAAATG

Sequence analysis of the initial PCR product obtained from the inverse PCR reaction revealed that the MMTV-Cre transgene was physically linked to a DNA fragment that included the K14 promoter linked to the agouti gene, followed by the poly A addition signal from the 3′end of the human growth hormone gene. This sequence was identical to the transgene termed the K14-agouti cassette originally described by Kucera et al [14]. This result was surprising because there was no mention of the use of the K14-agouti cassette in the original paper describing the generation of the MMTV-Cre mice [5]. We have recently discovered however, that the K14-agouti transgene was co-injected with the MMTV-Cre DNA to facilitate the identification of mice that displayed germ line integration of the transgene, since these mice display light yellow fur color in the background of black fur (L. Hennighausen, personal communication). Repeating the same approach by using two pairs of nested primers located in K14 agouti cassette confirmed these data and revealed that the DNA adjacent to the K14-agouti transgene was the MMTV-Cre transgene. No other mouse genomic DNA sequences were found after these two rounds of inverse PCR. These results suggest strongly that multiple MMTV-Cre cassettes are ligated to multiple K14-agouti cassettes in an alternating fashion. PCR analyses, using different PCR primer pairs that include one primer in the MMTV-Cre cassette and the other primer in the K14-agouti cassette, revealed that the MMTV-Cre cassette is ligated to the K14-aguoti cassette in a head-to-tail and tail-to-tail fashion (Figure 4B).

Three independent MMTV-Cre lines (A, D, and F) were initially generated by the Hennighausen lab [5] and they have been used in various studies of the mammary gland. Line A and line D [6], [20] are the more widely used founder lines in comparison to the number of studies that have utilized founder line F [21]. To confirm which MMTV-Cre line was used in our study, we performed PCR analysis using primer pairs that can distinguish the head-to-tail [MMTV-1R/human growth hormone polyadenylation sequence (GHA)-1R primer set] and tail-to-tail (RGI-1F/GHA-1R primer set) linkage of the MMTV-Cre and the K14-agouti cassettes (Figure 4B). We determined that the MMTV-Cre line used in our studies was line A because genomic DNAs from our MMTV-Cre line (Figure 4, lane 2), the MMTV-Cre line A from the Jackson laboratory (figure 4, lane 3), and a recently obtained DNA sample of line A from the Hennighausen lab (Figure 4, lane 5) all contain both the head-to-tail and tail-to-tail linkages. In contrast, genomic DNAs from line D (obtained from Jackson Laboratory) (Figure 4, lane 4) and line F (obtained from the Hennighausen lab) (Figure 4, lane 6) contain only the tail-to-tail linkage of the MMTV-Cre and the K14-agouti cassettes (Figure 4B). Interestingly, the PCR product of the tail-to-tail linkage from Line D (Figure 4B Bottom panel, lane 4) is ∼50 bp smaller than those products from Line A (lane 2,3,5) and Line F (lane 6). DNA sequencing confirms that the PCR product of the tail-to-tail linkage from Line D lacks 12 bp sequence in the 3′ end of the rabbit growth hormone intron (RGI) sequence and 40 bp sequence in the 3′end of the human growth hormone polyadenylation (GHA) sequence. Thus, by performing PCR analysis on genomic DNA using the two primer sets (MMTV-1R/GHA-1R for head-to-tail and RGI-1F/GHA-1R for tail-to-tail) (see Table 1 and Figure 4B), MMTV-Cre line A, D, and F can be distinguished from each other. Both the MMTV-Cre line A and line D mice available from the Jackson laboratory were originally donated by the Hennighausen lab. The MMTV-Cre line F generated by the Hennighausen lab is only available from the Mouse Models of Human Cancer Consortium.

## Discussion

These studies were initially undertaken to examine the role of Shp1, a protein tyrosine phosphatase, in mammary gland development. The observation that mice containing a single allele of Shp1 displayed a lactation defect (Figure 1) was of great interest since we did not anticipate that a 50% decrease in Shp1 protein expression in mammary epithelial cells would have such a dramatic effect. A careful review of the Shp1 literature, however, strongly suggested that this conclusion may not be correct. The naturally occurring Shp1 mutation observed in the motheaten (me) mice was discovered in 1975 [22], and heterozygous Shp1^me/+^ mice express only 50% of the Shp1 protein in all tissues. Shp1^me/+^ mice female mice are healthy, and no lactation defects have been reported or observed in Shp1^me/+^ female mice [22]. The absence of a lactation defect in the Shp1^me/+^ mice caused us to conclude that it was more likely that the MMTV-Cre transgene contributing to the lactation defect in these mice rather than lower amounts of the Shp1 protein. Unfortunately, the original paper describing the generation of the MMTV-Cre mice did not mention whether the MMTV-Cre female mice lactated normally [5].

In this study, we demonstrate that the MMTV-Cre line A mice [5], have a severe lactation defect. The defect is associated with reduced lobular-alveologenesis during pregnancy, impaired secretory activation as revealed by large cytoplasmic lipid droplets within mammary epithelial cells postpartum and the early onset of involution of mammary glands during early lactation. The MMTV-Cre line A has been widely used to study gene function in the mammary gland during pregnancy and lactation [23], [24], [25], [26] or tumorigenesis [27], [28]. The fact that the line A mice were used in these studies led us to assume that the lactation status of MMTV-Cre line A had been examined and determined to be normal. However, careful review of the literature involving MMTV-Cre lines generated by Wagner et al [5] indicate that the lactation status of these mice (line A, D, and F) has never been specifically described. Most importantly, our review of the studies that have utilized these MMTV-Cre lines indicated that either wild type mice or mice that were homozygous for the floxed alleles were generally used as the control mice. Based on our findings in this paper, it is important to re-examine conclusions drawn from previous studies using the MMTV-Cre line A mice [23], [24], [25], [26]. In addition, it is also important to examine whether MMTV-Cre lines D and F have lactation defects similar to what we have found in the line A mice. The situation is further confused by the fact that numerous published studies have not clearly indicated which line of the MMTV-Cre mice was utilized. It is possible that some authors may not recognize the differences among different MMTV-Cre lines generated by the Hennighausen laboratory [21], [29], [30], [31], [32]. The strategy that we have used to clone the integration site for the MMTV-Cre transgene has resulted in a PCR genotyping method that allows one to distinguish among the A, D, and F lines of the MMTV-Cre mice (Figure 4B), and this can be used to establish the identity of the lines in these published studies. In addition to the MMTV-Cre lines established by the Hennighausen laboratory, two other laboratories have generated independent MMTV-Cre lines [7], [8]. Both of these other lines show relatively specific deletion of the floxed genes in mammary epithelial cells; however, only Boussadia *et al* specifically stated that mammary gland development was normal during pregnancy and lactation [7], [8].

It is not clear why the MMTV-Cre line A dams display a lactation defect, and there are several possibilities could potentially explain this phenotype. First, the genetic background of the mice could play a role. The MMTV-Cre line A mice used in our studies had been backcrossed onto the FVB background more than 9 generations. The agouti locus was used as a marker for transgene integration in mice that have a black coat color such as C57BL6; the original MMTV-Cre mice were generated on the C57BL6 genetic background. A review of the literature indicates that the majority of published studies using the MMTV-Cre line A mice predominantly utilized mice on a mixed genetic background (i.e. mixture of two or three backgrounds such as C57BL6, 129Sv, and FVB) [23], [24], [25], [26]. Thus, it is possible that an allele important for lactation on the FVB background could result in the appearance of the lactation defect in MMTV-Cre line A mice only on this genetic background. While this manuscript was in preparation, a study by the Hennighausen lab indicated that MMTV-Cre line A mice on mixed genetic background comprising the 129Sv and C57BL6 strains also have a lactation defect [33]. In this study, pups nursed by line A dams weighed only ∼40% of controls at L15 [33]. Therefore, it is unlikely that the lactation defect described in our study is specific to mice on the FVB background. Second, the MMTV-Cre line A mice may express exceptionally high levels of Cre recombinase in the mammary gland during pregnancy, and it has been reported that acute high expression of Cre in mammary cells can inhibit cell growth via non-specific excision of DNA by Cre recombinase [4]. We think that this possibility is unlikely because our preliminary data show that the level of Cre mRNA expression in the pregnant mammary glands from our MMTV-Cre line A and BLG-Cre mice appears to be similar (data not shown), and BLG-Cre female mice do not exhibit a lactation defect [34]. Third, the integration of the MMTV-Cre transgene into the mouse chromosome may disrupt a gene important for lobuloalveolar growth and differentiation. Our inverse PCR studies were designed to identify the integration site for the transgene in the line A mice as described in Figure 4, however this approach revealed that the MMTV-Cre transgene is actually linked to the K14-agouti cassette in a repeated fashion, which limited our ability to identify the genomic integration site for the MMTV-Cre transgene. Finally, the presence of the K14-agouti cassette could also poise a potential problem in either mammary gland development or secretory activation. The product of the Agouti gene has been reported to inhibit lipolysis in human adipocytes [35], and thus expression of the K14-agouti cassette could potentially inhibit lipolysis in the mammary gland, or alternatively modify lipid biosynthesis in mammary epithelial cells resulting in the described lactation defect. Resolution of these different alternatives will require more extensive investigation and is likely to yield interesting results.

In conclusion, we find unexpectedly that MMTV-Cre line A, one of the three widely used MMTV-Cre transgenic mouse lines generated by the Hennighausen laboratory, exhibits a severe lactation defect. The existence of this lactation defect argues against using the MMTV-Cre line A mice to study gene function in mammary gland during pregnancy and lactation. In addition, we uncovered that the MMTV-Cre transgene is physically linked to the K14-agouit transgene in all of these MMTV-Cre mouse lines generated by the Hennighausen laboratory [5], [6]. Using this information, we have developed a PCR-genotyping method that allows these three different founder lines of MMTV-Cre mice to be readily distinguished. A recent study indicates that MMTV-Cre line D mice have a minor lactation defect, and that the MMTV-Cre line F mice have a more severe lactation defect than line A mice analyzed in the same study [33]. We advise researchers to take extreme caution when using one of these MMTV-Cre founder lines [5], [6] in their studies if they intend to evaluate secretory activation and/or lactation. It should also be readily apparent that mice expressing the MMTV-Cre transgene alone must be used as a control for all studies.

## Materials and Methods

### Ethical Statement

All animals were handled in accordance with protocols for the humane treatment of animals. This study was approved by the University of Colorado Denver Institutional Animal Use and Care Committee.

### Mice

MMTV-Cre mice (A) mice (mixed C57BL/6 and Sv129 genetic background) were obtained from L. Hennighausen (NIH) (Wagner et al 1997), and were backcrossed onto FVB background for more than 9 generations prior to use. Genotyping was conducted using primers as described (Wagner et al 1997). Male MMTV-Cre mice were mated with female FVB mice during backcrossing and strain maintenance. Shp1 floxed (fl) mice [13] were backcrossed onto FVB background for more than 9 generations. The primers for genotyping Shp1 floxed (fl) mice include SHCP 27 (5′-ACCCTCCAGCTCCTCTTC-3′), SHCP 29 (5′-TGAGGTCCCGGTGAAACC-3′), and SHCP 32 (5′-TGTTATGCATGTGTGTATCG-3′). The primer set, Cre-F and Cre-R, was used to genotype the MMTV-Cre mice (see Table 1 for sequence information). Six-week old Shp1 fl/+ (control), Shp1 fl/+; MMT-Cre, and MMTV-Cre female virgin mice were used in the study. All of the mice used in this study were hemizygous for the MMTV-Cre transgene. Mice were maintained in the Center for Comparative Medicine at the University of Colorado, Anshutz Medical Campus, an AAALAC-approved facility, and provided chow and water *ad libitum*.

### Quantitation of lactation competency

Lactation day 1 (L1) was defined as the day upon which pups were born. All litters from the Shp1 fl/+ (control), Shp1 fl/+; MMT-Cre, and MMTV-Cre dams were normalized to 6 pups/litter for each nursing dam on L2. Pups in each normalized litter were weighted at L5, L8, and L10. The average weight of each litter was determined and plotted versus the day of lactation; this method has been used to accurately determine the lactation competency of lactating dams [15], [36]. The significance of the pup weight difference was determined by analysis using unpaired student Two-tailed t-test.

### Antibodies and reagents

Antibodies to phosphorylated Stat3 (Y705) and phosphorylated Stat5 (Y694) were purchased from Cell Signaling (Beverly, MA). Rabbit polyclonal antibodies to Stat3 and Stat5 were from Santa Cruz Biotechnology (Santa Cruz, CA). Guinea Pig polyclonal antibodies to cytokeratin-8/18 were from Fitzgerald Industries International (www.fitzgerald-fii.com). Anti-beta-actin mouse monoclonal antibody was from Sigma Chemical Company. Restriction enzymes and modification enzymes T4 DNA polymerase, T4 DNA ligase and Phire Hot Start DNA polymerase were from New England Biolab (Ipswich, MA). SignalStain™ Cleaved Caspase3 (Asp175) Immunohistochemistry (IHC) detection kit was purchased from Cell Signaling (Beverly, MA). DNA primers were synthesized by Invitrogen. Genomic DNAs for MMTV-Cre line A and MMTV-Cre line D were purchased from Jackson Laboratory (Bar Harbor, Maine). Recently prepared genomic DNAs for MMTV-Cre line A and line F were also kindly provided by Drs. G.W.Robinson and L. Hennighausen (NIH).

### Analysis of mammary gland histology and apoptosis

The number 4 mammary glands were dissected and fixed overnight in 10% neutral buffered formalin. The fixed mammary tissue was embedded in paraffin, 4 µm sections prepared, and stained with hematoxylin and eosin by the Pathology Core Laboratory at University of Colorado Cancer Center. For whole-mount analysis, the dissected mammary glands were placed in histology cassettes, and fixed overnight in 10% neutral buffered formalin. The fixed mammary glands were defatted in acetone (3 washes, one hour/wash), rehydrated through graded alcohols, and stained overnight with carmine alum solution (0.2% carmine, 0.5% aluminum potassium sulfate in distilled water). The whole mounts were then rinsed in running water, rinsed in acidic 50% ethanol (pH 1.25), dehydrated in graded ethanols, prior to placing in xylene. Stained glands were mounted with Permount (Fisher Scientific). Apoptotic cells in mammary glands from lactation day 10 mice were quantitated by staining thin sections with the cleaved caspase3 IHC detection kit (Cell Signaling) according to the manufacturer's protocol. Cleaved caspase3 positive cells were quantified by counting caspase3-stained cells in ten high-power fields (40 x objective lens) with equivalent density of mammary epithelial cells. The average number of caspase3 positive cells per 40 x field is used for comparison +/- standard deviation. Mammary glands were analyzed from two individual mice for each genotype.

### Biochemical analysis of mammary glands

Number 4 mammary glands were dissected, flash frozen in liquid nitrogen, and the tissue disrupted by sonication with a Polytron (Brinkmann Instruments) in mammary gland lysis buffer [(50 mM Tris pH 7.4, 150 mM NaCl, 2 mM EDTA, 50 mM NaF, 1% Triton X-100, 1% DOC, 0.1% SDS, 1 mM DTT, 5 mM sodium orthovanadate, supplemented with 100 µg/ml PMSF, and Complete protease inhibitor cocktail (Roche Applied Sciences, Indianapolis, IN)]. Lysates were clarified by centrifugation and soluble protein concentration was measured by Bradford method. Equal amounts of protein samples were resolved by SDS-PAGE, transferred to Immobilon-P membrane (Millipore Inc.), immunoblotted using the appropriate primary and secondary antibodies, and immunoreactive bands detected by enhanced chemiluminescence.

### Identification of the K14-agouti cassette linked to MMTV-Cre transgene by Inverse PCR

Livers from wild type and MMTV-Cre mice were digested in buffer containing 2 mg/ml protease K, 10 mM Tris pH 8.0, 25 mM EDTA, 0.5% SDS, 100 mM NaCl at 55°C overnight. DNA was extracted with Phenol/Chloroform, ethanol precipitated, and resuspended in distilled water. Purified genomic DNAs were digested with restriction enzymes and blunt-ended fragments generated by treatment with T4 DNA polymerase, after which circular DNA molecules were generated by self-ligation with T4 DNA ligase for use as templates for inverse PCR. After 1^st^ round PCR amplification using the primer set RGI-1F/RGI-1R and the Phire Hot Start DNA polymerase, the product was diluted 100-fold and used as template in 2^nd^ round nested PCR amplification using the primer set RGI-2F/RGI-2R. The amplified PCR products were purified for sequencing. Most of the PCR products were generated by amplification of tandem repeats of the MMTV-Cre transgene. To identify PCR products that included the human growth hormone polyA addition signal present in the K14-Agouti transgene, genomic DNA was digested with combinations of HincII/KpnI or HincII/XbaI prior to PCR amplification [14]. The MMTV-1R/GHA-1R primer set was used to identify the head-tail linkage of the MMTV-Cre and the K14-agouti cassettes. The RGI-1F/GHA-1R primer set was used to identify the tail-tail linkage of the MMTV-Cre and the K14-agouti cassettes. TheK14P-3R/GFA-1F primer set was used to identify the presence of the K14-agouti cassette. The sequences of all primers used in this study are listed in Table 1.
